# Expression Profiles of Epithelial-Mesenchymal Transition-Associated Proteins in Epithelial Ovarian Carcinoma

**DOI:** 10.1155/2014/495754

**Published:** 2014-04-01

**Authors:** Mi-Kyung Kim, Min A Kim, Haeryoung Kim, Yong-Beom Kim, Yong-Sang Song

**Affiliations:** ^1^Biomedical Science Project, Brain Korea 21 Program for Leading Universities & Students, Seoul National University, Seoul, Republic of Korea; ^2^Department of Pathology, Seoul National University College of Medicine, Seoul, Republic of Korea; ^3^Department of Pathology, Seoul National University Bundang Hospital, Seongnam, Republic of Korea; ^4^Department of Obstetrics and Gynecology, Seoul National University Bundang Hospital, Seongnam, Republic of Korea; ^5^Department of Obstetrics and Gynecology, Seoul National University College of Medicine, 101 Daehak-ro, Jongno-gu, Seoul 110-744, Republic of Korea; ^6^Cancer Research Institute, Seoul National University College of Medicine, Seoul, Republic of Korea; ^7^WCU Biomodulation Major, Department of Agricultural Biotechnology, Seoul National University, Seoul, Republic of Korea

## Abstract

Epithelial-mesenchymal transition (EMT) has been suggested to contribute to tumor progression and acquisition of therapeutic resistance. To assess the clinical significance of EMT-associated proteins, we evaluated the expression of Snail and Slug, the key regulators of EMT, in the primary ovarian cancer samples (*n* = 103) by immunohistochemistry. Snail was differentially expressed according to the histologic subtype (*P* = 0.001) and was predominantly expressed in serous and endometrioid types. In the serous and endometrioid adenocarcinomas, the expression of Snail remained high across the stage and grade, suggesting its role in the early phase of carcinogenesis. However, the expression of Snail and Slug was not related to chemoresistance and poor prognosis and did not serve as independent predictive or prognostic marker.

## 1. Introduction

Ovarian cancer is the seventh leading cause of cancer deaths in women worldwide and is the most lethal gynecologic malignancy [1]. Despite advances in surgery and chemotherapy, overall cure rate has remained approximately 30%. The poor clinical outcome mainly comes from the high percentage of cases being diagnosed at an advanced stage and the frequent emergence of chemoresistance. Recent evidence has suggested that epithelial-mesenchymal transition (EMT) may contribute to tumor invasion, metastasis, and acquisition of therapeutic resistance [2]. The term EMT refers to a complex molecular and cellular program that involves the loss of cell adhesion and acquisition of migratory and mesenchymal features. EMT plays a key role in normal physiologic processes during embryogenesis and wound healings, but it has also been recognized in the pathogenesis of cancer. During carcinogenesis, EMT is not only responsible for acquiring and maintaining mesenchymal phenotypes such as invasiveness and resistance to apoptosis but also confers stem cell-like characteristics upon cancer cells [3]. In addition, the expression of EMT signaling pathways has been associated with poor prognosis in various epithelial cancers, including breast, pancreas, prostate, and ovarian cancer [4].

The Snail family members, Snail (*SNAI1*) and Slug (*SNAI2*), are key regulators of EMT and directly repress the transcription of E-cadherin, a cell adhesion molecule. In epithelial ovarian cancer, the expression of these two transcriptional repressors along with the loss of E-cadherin has been shown to be related to tumor progression and sometimes poor prognosis [5–7]. In most of these studies, however, the expression of Snail and Slug has not been evaluated and compared between different subtypes of ovarian cancer which are now considered as different disease entities with distinct biomarker expression profiles [8, 9]. In addition, it has recently been proposed that ovarian cancer can be divided into two groups based on genetic changes: low-grade (type I) and high-grade (type II) ovarian cancer [10, 11]. Type I cancers progress through a stepwise mutation process and frequently harbor* PTEN*,* KRAS*, and* BRAF* mutations. In contrast, type II cancers are characterized by frequent* TP53* mutation and progress through genetic instability without identifiable precursor lesions.

Therefore, in this study, we analyzed the differential expression of Snail and Slug according to the histologic subtype by immunohistochemistry. The p53 expression, which has been shown to be frequently aberrant in serous type, was also assessed to evaluate the correlation between p53 and EMT-related proteins. In addition, we explored the predictive and prognostic significance of Snail and Slug in epithelial ovarian cancer.

## 2. Materials and Methods

### 2.1. Patients

A total of 103 patients who had undergone primary debulking surgery for stages I–IV epithelial ovarian cancer between 2003 and 2009 at Seoul National University Bundang Hospital were included in the study analysis after obtaining approval from the institutional review board. Exclusion criteria included patients who received neoadjuvant chemotherapy before surgery because chemotherapy might be able to affect the proportion of chemoresistant tumor cells and change the expression level of EMT proteins [12, 13]. Patients with recurrent or nonepithelial ovarian cancer were also excluded. Clinicopathologic data, including age, the international federation of gynecology and obstetrics (FIGO) stage, surgical procedures, the extent of residual disease, histologic subtype, grade, adjuvant chemotherapy, and survival outcomes, were evaluated by reviewing medical charts and pathologic records.

### 2.2. Tissue Samples

Tissue microarrays (TMAs) were constructed from core biopsies (diameter 2 mm) of formalin-fixed paraffin-embedded primary ovarian cancer specimens using a trephine apparatus (SuperBioChips Laboratories, Seoul, Korea). Three core biopsies were taken from each individual specimen [14].

### 2.3. Immunohistochemistry

To detect Snail and Slug-specific immunoreactivity, sections (4 *μ*m) from array blocks were treated as follows: after standard pressure-cooker-based antigen retrieval with citric acid (pH 6.0) pretreatment, sections were incubated with 1% horse serum in Tris-buffered saline for 3 minutes. The sections were incubated with either a rabbit polyclonal anti-Snail antibody (1 : 800) (ab17732; Abcam, Cambridge, UK) or a rabbit polyclonal anti-Slug antibody (1 : 100) (ab27568; Abcam). Both antibodies were detected using the polymer for 8 minutes and DAB substrate for 10 minutes (Leica Bond-Max Autostainer; Leica, Wetzlar, Germany). For p53 immunoreactivity, similar techniques were applied using a mouse monoclonal anti-p53 antibody (1 : 100) (M7001; DAKO, Carpinteria, USA) as a primary antibody.

Immunostaining of Snail and Slug was evaluated by two independent observers (K.M.K. and K.M.A.) for both the percentage of positive cells and staining intensity from 1 to 3 (1 weak, 2 moderate, and 3 strong). Since three cores were taken from each tumor, the average value was used for the study analysis [14]. Snail expression was mainly localized to the nucleus with weak cytoplasmic staining, and Slug was expressed in cytoplasm of tumor cells. In general, staining for Snail was more intense than that for Slug. For further statistical analysis, Snail and Slug expression was categorized into two groups: high expression, when >50% of tumor cells showed moderate-to-strong intense staining, and low expression, when ≤50% of tumor cells were positive [15, 16].

Nuclear expression of p53 was recorded as follows: completely negative, any staining in ≤50% of tumor cells, or moderate-to-intense staining in >50% of tumor cells [17].

### 2.4. Statistical Analysis

The differences in clinicopathologic variables according to the immunoreactivity for Snail and Slug were evaluated using chi-square test or Student's *t*-test accordingly. Survivals were also evaluated and compared using Kaplan-Meier method and log-rank test. Progression-free survival (PFS) was defined as the time interval from surgery to the first evidence of recurrence or death from any cause, whichever occurred first. Overall survival (OS) was defined as the time from surgery to death from any cause. A *P* value of less than 0.05 was considered to indicate statistical significance, and all tests were two-sided. The statistical analysis was performed using SPSS for Windows (version 19.0; SPSS Inc., Chicago, IL).

## 3. Results

### 3.1. Snail/Slug Expression and Clinicopathologic Variables

Of the 103 cases with epithelial ovarian cancer, serous type was the most frequently diagnosed histologic subtype (59.2%), followed by mucinous (16.5%), clear cell (12.6%), and endometrioid type (9.7%). Most of the patients were diagnosed with stage I (34.0%) and stage III (46.6%) diseases. The majority of patients (88.5%) received platinum-based chemotherapy after the debulking surgery.

**Figure 1:**
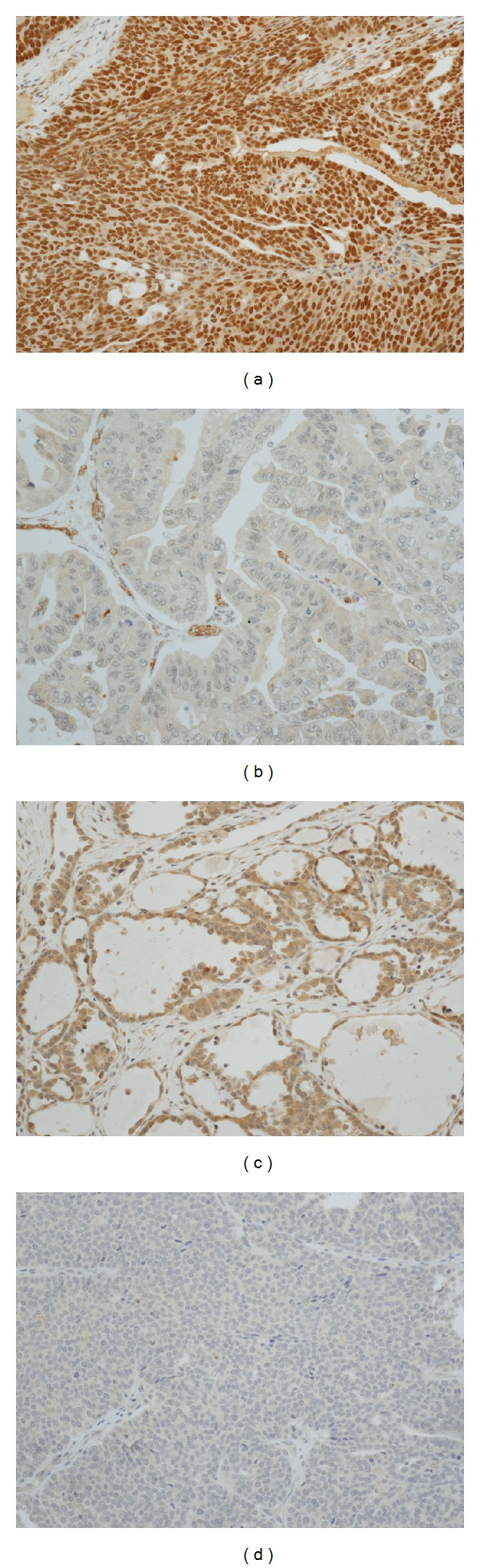
Immunohistochemical staining for Snail and Slug. (a) Snail-positive in serous carcinoma, (b) Snail-negative in mucinous carcinoma, (c) Slug-positive in clear cell carcinoma, and (d) Slug-negative in serous carcinoma. All figures are at 200× magnification.

Snail was widely expressed (96.1%) and 81.6% of the cases showed high Snail expression. Slug was also expressed in the majority of tumors (91.3%), but high Slug expression was shown in 28.2% of the cases.  Figure 1 shows the representative immunohistochemical findings. Snail expression was significantly higher in serous and endometrioid subtype than in mucinous or clear cell type (*P* = 0.001; Table 1). Snail expression also showed a tendency to correlate with high-grade lesions (*P* = 0.048). However, other clinical variables, such as FIGO stage, lymph node metastasis, peritoneal seeding, and residual disease status, were not associated with Snail expression. Slug expression was not significantly associated with Snail expression (*P* = 0.058), and it was not associated with clinicopathologic variables, including histologic subtype (Table 1).

When the analysis of Snail expression was limited to serous adenocarcinomas, Snail expression remained high across the stage and grade (Table 2).

### 3.2. Differential Expression of Snail According to the p53 Expression

p53 was differentially expressed according to the histologic subtype of ovarian cancer (*P* < 0.001). The aberrant p53 expression, which was defined as completely negative or >50% expression [18], was significantly higher in the serous type compared to the mucinous or endometrioid subtype (86.9% versus 41.2% or 30.0%). When assessing the relationship between p53 and EMT-related proteins in serous adenocarcinomas, p53 expression was not significantly correlated to the Snail and Slug expression (*P* = 0.537 and *P* = 0.132, resp.; Table 3).

### 3.3. Snail/Slug Expression and Survival Outcomes

In serous adenocarcinomas, survival outcomes failed to show statistically significant differences between Snail^low^ and Snail^high^ population. Although there was a trend of worse PFS in Snail^high^ patients (2-year PFS, 48.1% in Snail^high^ versus 53.3% in Snail^low^), the difference was not statistically significant (*P* = 0.285). Overall survivals also failed to show a significant difference according to the Snail expression (*P* = 0.382). Similarly, Slug expression was not associated with survival outcomes. In addition, when the platinum resistance was defined as recurrent within 6 months after the last chemotherapy, it was not associated with Snail expression (*P* = 0.594; Table 2).

## 4. Discussion

In the present study, we demonstrated that Snail was differentially expressed according to the histologic subtype and was highly expressed in the serous and endometrioid carcinomas. Although the expression profile of Snail was found to be subtype-specific, it failed to serve as an independent predictive or prognostic marker. The finding of differential expression of Snail according to the histologic subtype suggests that Snail might have different roles in tumor progression depending on the subtype of ovarian cancer. In addition, the high expression of Snail in the early stage serous carcinomas may suggest the potential role of Snail in the early phase of carcinogenesis.

Snail has been associated with poor clinical outcomes in various tumor types, including ovarian cancer, through induction of EMT which is responsible for metastasis and acquisition of therapeutic resistance. In epithelial ovarian cancer, Snail and Slug were shown to have distinct roles in metastasis and cancer cell survival [19, 20]. In addition, Snail and Slug were shown to contribute to the development of resistance to radiation and chemotherapy through overcoming p53-mediated apoptosis and acquisition of stem-like characteristics in ovarian cancer cells [13]. Snail was also shown to be highly expressed in advanced stage and metastatic lesions [5, 21]. However, most of these studies did not evaluate the differential expression of EMT proteins according to the different subtypes. The present study included the primary ovarian cancer specimens with various histologic subtypes, which enabled the comparison of the distribution of histologic subtypes and survival outcomes according to the expression of EMT proteins more relevantly and demonstrated that the Snail and Slug were not independently related to survival outcomes as well as response to chemotherapy.

*TP53* mutation, which is represented by aberrant p53 expression, is present in almost all cases of high-grade serous ovarian cancer (96%) [22]. In the present study, aberrant p53 expression was also frequently observed in serous type (86.9%). However, in serous adenocarcinomas where both p53 and Snail demonstrated aberrant expression commonly, Snail expression was not affected by p53 status. This might suggest that p53 and Snail have potentially different roles in ovarian carcinogenesis.

The immunopositivity of Snail in this study was much higher than the results of previous studies which reported the positive rate as 23–37.5% [6, 7]. In addition, some studies reported cytoplasmic staining of Snail rather than nuclear staining which is considered to be an active form [5, 21]. These discrepancies might be originated from the different antibodies used and the nonstandardized evaluation of staining. Our finding of widespread nuclear expression of Snail, however, is consistent with the previous study which demonstrated that Snail mRNA and protein expression were detected in almost all primary ovarian tumor specimens (93% and 100%, resp.) [23].

In the present study, the evaluation of the underlying mechanisms was limited due to the immunohistochemical analysis. In addition, the retrospective study design might cause selection biases. However, our finding of the differential distribution of tumor cells overexpressing Snail according to the histologic subtype may provide useful information regarding the patient selection for targeted therapy against EMT pathways.

In conclusion, we demonstrated that Snail expression was predominant in serous and endometrioid adenocarcinomas by immunohistochemistry. Snail and Slug overexpression, however, did not correlate with poor clinical outcomes. Our study set the stage for future studies investigating the differential roles of EMT according to the different histologic subtypes, which may provide potential therapeutic targets against cancer progression and metastasis.

## Figures and Tables

**Table 1:** Expression of Snail and Slug according to the clinicopathologic variables (*N* = 103).

Variables	Snail^low^, *n* (%)	Snail^high^, *n* (%)	*P* value	Slug^low^, *n* (%)	Slug^high^, *n* (%)	*P* value
Stage						
I	6 (17.1)	29 (82.9)	0.185	22 (62.9)	13 (37.1)	0.402
II	1 (9.1)	10 (90.9)		8 (72.7)	3 (27.3)	
III	8 (16.7)	40 (83.3)		36 (75)	12 (25)	
IV	4 (44.4)	5 (55.6)		8 (88.9)	1 (11.1)	
Histology						
Serous	5 (8.2)	56 (91.8)	0.001	45 (73.8)	16 (26.2)	0.097
Mucinous	7 (41.2)	10 (58.8)		8 (47.1)	9 (52.9)	
Endometrioid	1 (10)	9 (90)		9 (90)	1 (10)	
Clear cell	6 (46.2)	7 (53.8)		10 (76.9)	3 (23.1)	
Others	0 (0)	2 (100)		2 (100)	0 (0)	
Grade						
1	7 (33.3)	14 (66.7)	0.048	12 (57.1)	9 (42.9)	0.215
2	8 (21.6)	29 (78.4)		29 (78.4)	8 (21.6)	
3	4 (8.9)	41 (91.1)		33 (73.3)	12 (26.7)	
LN metastasis						
No	14 (18.4)	62 (81.6)	0.927	53 (69.7)	23 (30.3)	0.483
Yes	5 (19.2)	21 (80.8)		20 (76.9)	6 (23.1)	
Peritoneal seeding						
No	10 (16.4)	51 (83.6)	0.804	44 (72.1)	17 (27.9)	0.990
<2 cm	2 (20.0)	8 (80.0)		7 (70.0)	3 (30.0)	
>2 cm	7 (21.9)	25 (78.1)		23 (71.9)	9 (28.1)	
Residual tumor						
<1 cm	12 (17.6)	56 (82.4)	0.667	45 (66.2)	23 (33.8)	0.103
>1 cm	7 (21.2)	26 (78.8)		27 (81.8)	6 (18.2)	

LN: lymph node.

**Table 2:** Expression of Snail according to the clinicopathologic variables in serous adenocarcinomas (*n* = 61).

Variables	Snail^low^, *n* (%)	Snail^high^, *n* (%)	*P* value
Stage			
I/II	1 (5.3)	18 (94.7)	0.574
III/IV	4 (9.5)	38 (90.5)	
Grade			
1	0 (0)	5 (100)	0.591
2	3 (12)	22 (88)	
3	2 (6.5)	29 (93.5)	
LN metastasis			
No	4 (9.3)	39 (90.7)	0.666
Yes	1 (5.9)	16 (94.1)	
Responses			
CR/PR	5 (10.2)	44 (89.8)	0.561
SD/PD	0 (0)	3 (100)	
Platinum sensitivity*			
Sensitive	4 (12.5)	28 (87.5)	0.594
Intermediate	1 (7.1)	13 (92.9)	
Resistant	0 (0)	6 (100)	

LN: lymph node; CR: complete response; PR: partial response; SD: stable disease; PD: progressive disease.

*Platinum sensitivity was defined according to the time interval from the date of last chemotherapy cycle to the first evidence of recurrence; sensitive when the interval was >12 months, intermediate when the interval was >6 months and <12 months, and resistant when the interval was <6 months.

**Table 3:** Expression of p53 according to Snail and Slug expression in serous adenocarcinomas (*n* = 61).

	p53 negative *n* (%)	p53 <50% *n* (%)	p53 >50% *n* (%)	*P* value
Snail				
Negative	2 (40.0)	0 (0)	3 (60.0)	0.537
Positive	13 (23.2)	8 (14.3)	35 (62.5)	
Slug				
Negative	14 (31.1)	5 (11.1)	26 (57.8)	0.132
Positive	1 (6.2)	3 (18.8)	12 (75.0)	
